# Analysis of Electronic Properties from Magnetotransport Measurements on Ba(Fe_1−x_Ni_x_)_2_As_2_ Thin Films

**DOI:** 10.3390/ma13030630

**Published:** 2020-01-31

**Authors:** Ilya Shipulin, Stefan Richter, Aleena Anna Thomas, Kornelius Nielsch, Ruben Hühne, Victor Martovitsky

**Affiliations:** 1 V.L. Ginzburg Center for High-Temperature Superconductivity and Quantum Materials, P.N. Lebedev Physical Institute of the Russian Academy of Sciences, Moscow 119991, Russia; 2 Institute for Metallic Materials, Leibniz IFW Dresden, 01069 Dresden, Germany; 3 School of Sciences, TU Dresden, 01062 Dresden, Germany

**Keywords:** iron-based superconductors, thin films, pulsed laser deposition, electronic properties

## Abstract

We performed a detailed structural, magnetotransport, and superconducting analysis of thin epitaxial Ba(Fe_1−x_Ni_x_)_2_As_2_ films with Ni doping of x = 0.05 and 0.08, as prepared by pulsed laser deposition. X-ray diffraction studies demonstrate the high crystalline perfection of the films, which have a similar quality to single crystals. Furthermore, magnetotransport measurements of the films were performed in magnetic fields up to 9 T. The results we used to estimate the density of electronic states at the Fermi level, the coefficient of electronic heat capacity, and other electronic parameters for this compound, in their dependence on the dopant concentration within the framework of the Ginzburg–Landau–Abrikosov–Gorkov theory. The comparison of the determined parameters with measurement data on comparable Ba(Fe_1−x_Ni_x_)_2_As_2_ single crystals shows good agreement, which confirms the high quality of the obtained films.

## 1. Introduction

Over the past 10 years after the discovery of superconductivity at about 26 K in LaFeAs(O,F) [1], significant research efforts have been devoted to identifying other superconducting iron-based compounds and studying their functional properties. This search led to the discovery of several new families of iron-based superconductors [2,3,4]. Among them is the so-called “122” family, which has the composition *AE*Fe_2_As_2_ (*AE* = Ba, Sr, Ca). The structure of these 122 compounds allows for a number of possible atomic substitutions resulting in a superconducting state. This triggered extensive studies on these materials due to this structural flexibility and their beneficial superconducting characteristics, such as high critical fields and low anisotropy, relatively simple processing, and sufficient stability in a standard environment [4,5,6].

Within the last few years, significant progress was made in the field of single crystal growth of 122-based materials with isovalent, hole, or electron doping, which made it possible to study the thermodynamic and transport properties of these compounds [6,7]. At the same time, the preparation of high-quality epitaxial thin films of 122 materials resulted, at least for the BaFe_2_As_2_-based compounds, in properties comparable to single crystals (for examples, see recent reviews on this topic [8,9]). Such films are of particular interest for the study of both fundamental and applied questions [7]. In particular, thin films were used in various fundamental investigations, such as for the evaluation of magnetotransport properties, including the study of quantum oscillations, optical measurements in a wide frequency range, studies of tunneling spectroscopy, muon spin relaxation (µSR) studies, measurements of penetration depth and critical current density, studies of pinning mechanisms, etc. [10,11,12,13,14,15].

In our previous work [16], we reported on the growth of high-quality epitaxial Ba(Fe_1−x_Ni_x_)_2_As_2_ thin films with different nickel concentrations, as well as on their basic superconducting properties. Based on these studies, we evaluated the quality (i.e., the spatial uniformity) of selected films in more detail by sophisticated X-ray measurements, as well as by determining the temperature dependence of the magnetic susceptibility. The general aim of this extended investigation was to study the main electronic characteristics of Ba(Fe_1−x_Ni_x_)_2_As_2_ films with different nickel concentrations within the framework of the Ginzburg–Landau–Abrikosov–Gorkov (GLAG) theory [17,18] based on the measurements of their superconducting and magnetotransport properties. The results are compared afterwards to the data of corresponding parameters measured on Ba(Fe_1−x_Ni_x_)_2_As_2_ single crystals.

## 2. Experimental Section

### 2.1. Synthesis

Ba(Fe_1−x_Ni_x_)_2_As_2_ targets with a nominal Ni content of x = 0.05 and 0.08 were synthesized as described in detail in [16]. The resulting cylindrical pellets were used as target material for pulsed laser deposition (PLD) in an ultra-high vacuum setup applying a Coherent KrF excimer laser at a repetition rate of 7 Hz. The base pressure of the chamber was below 1 × 10^−8^ mbar. During deposition in vacuum we observed the pressure increase to a level of about 2 × 10^−7^ mbar. Polished CaF_2_ (001) single crystalline substrates were heated to a temperature of 750 °C prior to deposition. The target was scanned during deposition to ensure a uniform film thickness of about 100 nm. The film growth was monitored in situ, using reflection high-energy electron diffraction (RHEED, Staib Instruments, Langenbach, Germany). The observed streaks in the diffraction pattern indicate a smooth and epitaxial growth.

### 2.2. Characterization

In order to evaluate the structural properties of the deposited films in more detail compared to the previous study, high-resolution X-ray diffraction (XRD) was performed in a Panalytical X’PERT PRO diffractometer (Almelo, The Netherlands) utilizing pure Cu-K_α1_ radiation. The electronic transport properties of the films were determined in a physical property measurement system (PPMS, Quantum Design, San Diego, CA, USA) applying magnetic fields of up to 9 T along the c-axis of the crystal structure.

## 3. Results and Discussion

### 3.1. Structural Characterization

**Figure 1:**
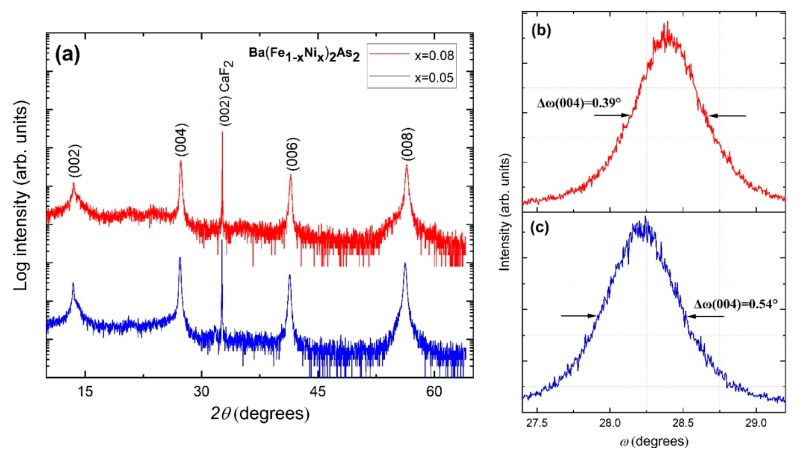
XRD patterns for Ba(Fe_1−x_Ni_x_)_2_As_2_ thin films grown on CaF_2_. (**a**) Standard *θ*–2*θ* XRD scans; rocking curves with determined full width at half maximum (FWHM) values of the (004) reflection for films with a nominal Ni content of: (**b**) x = 0.05 and (**c**) x = 0.08.

The XRD pattern in Figure 1a indicates a preferential *c*-axis-oriented growth as only (00ℓ) peaks are present in the *θ*–2*θ* scan for the Ni-doped Ba122 phase, as well as the single-crystal substrate. More detailed diffraction studies showed that the Ba(Fe_1−x_Ni_x_)_2_As_2_ thin films have a single-crystal-like structure with a parallel arrangement of the [1] axes of the layer and the CaF_2_ substrate. Moreover, the layers are arranged perpendicular to the growth surface, with a 45° in-plane rotation of the Ba122 [100] axis towards the similar CaF_2_ axis, in accordance with the smallest mismatch between the lattice parameters for the film (*a_L_*) and the substrate (*a_S_*), i.e., *a_L_* × √2 ≈ *a_S_*. To determine the *c*-axis mosaicity of the film, rocking curves were measured using the Ba122 (004) peak as shown in Figure 1b,c. The full width at half maximum (FWHM), Δω, of the rocking curves were 0.39° and 0.54° for the x = 0.05 and 0.08, respectively. These values indicate that our films have a good out-of-plane crystalline quality, which is only slightly inferior if compared with Ba122 single crystals (Δω = 0.18°) [6]. For Ba(Fe_1−x_Ni_x_)_2_As_2_ single crystals, the *c*-axis lattice parameter decreases with *c* = (13.0275 − 0.6x) Å [19] resulting in values *c*_(x=0.05)_ = 12.9975 Å and *c*_(x=0.08)_ = 12.9795 Å for the compositions studied in our work. The lattice parameter along the *c* axis is noticeably larger in our films compared to the single crystal values, but it also decreases with increasing nickel concentration, resulting in *c*_(x=0.05)_ = 13.0405 Å and *c*_(x=0.08)_ = 13.016 Å, respectively. 

The lattice parameter in the basal plane increases for single crystals from *a*_0_ = 3.9590 Å in undoped BaFe_2_As_2_ to 3.9606 Å in Ba(Fe_0.96_Ni_0.04_)_2_As_2_ [19], resulting in a relation of *a* = *a*_0_ *+* 0.08*x*. This results in a value of *a_(x =_*_0.05)_ = 3.963 Å and *a_(x =_*_0.08)_ = 3.965 Å for the compositions discussed here. The determined lattice parameters for our films are smaller compared to the single crystal values, with *a_(x_*_*=* 0.05*L)*_ = 3.952 Å and *a_(x =_*_0.08*L)*_ = 3.954 Å, but they increase with nickel concentration in a similar way. Thus, the unit cell of Ba(Fe_1−x_Ni_x_)_2_As_2_ epitaxial layers undergoes an additional tetragonal compression. All lattice parameters are summarized in Table 1. 

To discuss the magnitude of the tetragonal distortion for both doping values more quantitively, one can compare the difference of the measured in-plane lattice parameters between the film and single crystals to the values for the completely relaxed case, i.e., the difference between the substrate and the single crystal parameters. Therefore, we used the formula (*a_x_* − *a_xL_*)/(*a_x_* − *a_s_*) × 100%, where *a_x_* and *a_xL_* are the lattice parameters of a single crystal and the epitaxial layer of the same composition, and *a_s_* = *a_CaF_*_2_/√2 = 3.8634 Å is the substrate parameter along the [110] direction. The resulting value of about 11% is more or less independent from the Ni content, i.e., both films have a similar amount of strain included in the structure. The observed strain might arise from the differences in the thermal expansion coefficients between the film and the CaF_2_ substrate as already discussed previously [9,20].

### 3.2. Magnetotransport Characterization

**Figure 2:**
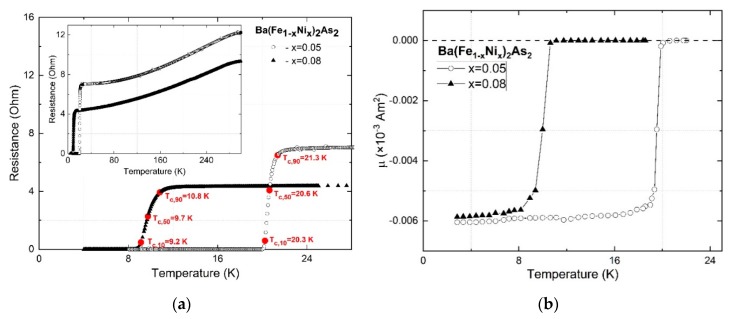
Temperature dependence of the resistance without a magnetic field (**a**) as well as susceptibility (**b**) for Ba(Fe_1−x_Ni_x_)_2_As_2_ films with x = 0.05 and 0.08.

Figure 2a summarizes the temperature dependence of the resistance for Ba(Fe_1−x_Ni_x_)_2_As_2_ films with x = 0.05 and 0.08 without an external magnetic field. The superconducting transition temperatures (*T_c_*), determined with a 90% criterion of the normal state resistance, were 21.3 K and 10.8 K for a nominal nickel content of x = 0.05 and 0.08, respectively. Figure 2b shows the magnetic susceptibility measured with zero field cooling (ZFC) in an external field of 10^−3^ T applied along the *c* axis. 

The ZFC data prove a sharp diamagnetic signal. The *T_c_* were determined from the onset of the diamagnetic transition temperature to be around 21.1 K and 10.3 K for x = 0.05 and 0.08, respectively, which fits well with the transport data. It is also worth noting that the obtained films show a *T_c_* which is slightly higher compared to published data for single crystals [6]. This may be due to the compressive strain induced by the CaF_2_ substrate [20,21]. The resistivity of the studied films are 0.211 mΩ·cm and 0.182 mΩ·cm for nickel concentrations of x = 0.05 and 0.08, respectively, which fits well with the resistivity measured on single crystals [22].

In our previous paper [16], we reported that *R*(*T*) has a quadratic dependence of resistance on the temperature of Ba(Fe_1−x_Ni_x_)_2_As_2_ thin films, which reflects the crucial contribution of electron-electron interaction to the scattering processes in these systems. Moreover, a similar dependence of the *R*(*T*) was observed in the overdoped phase of cuprate high-temperature superconductors (HTSCs), which is associated with strong interelectron correlations.

### 3.3. Electronic Structure Characterization

**Figure 3:**
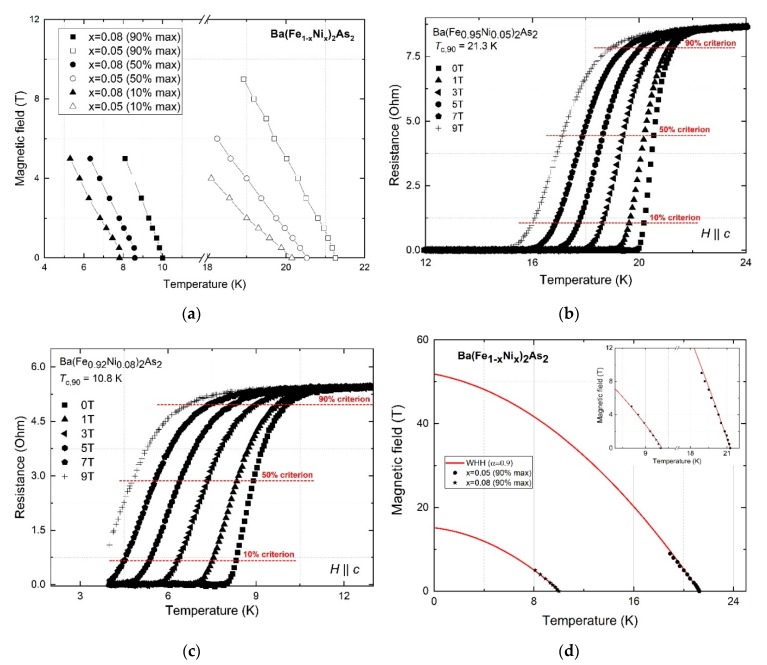
Temperature dependence of the magnetic field (**a**) and resistance in the applied magnetic field (**b,c**), as well as Werthamer–Helfand–Hohenberg (WHH) fitting with paramagnetic effect (**d**) for Ba(Fe_1−x_Ni_x_)_2_As_2_ films with x = 0.05 and 0.08. The complete dataset of the *R*(*T*,*B*) measurements is shown in a previous paper [16].

An algorithm was developed in the work of Golovashkin et al. [17] and Orlando et al. [18] for determining the electronic properties of typ Ⅱ superconductors using the results of magnetotransport measurements and the temperature dependence of the critical magnetic field *H_c_*_2_. This algorithm is based on the results of the GLAG theory and doesn’t contain any fitting parameters. We used this approach to determine the electronic properties of the obtained films. The critical magnetic fields *H_c_*_2_(0) for the obtained films were estimated using the Werthamer–Helfand–Hohenberg (WHH) model [23]. For comparison, the paramagnetic limit of *H_p_*(0) was calculated using the equation for a gap of the form Δ = 2.6 *k_B_T_c_*, which is far more consistent with experiments for pnictides [24]. The values of *dH_c_*_2_*(T)/dT* were evaluated with a 90% criterion. The temperature dependencies of the magnetic field are shown in Figure 3a–c for different criteria of *T_c_*.

For optimal doped Ba(Fe_0.95_Ni_0.05_)_2_As_2_ film, a value of $-dH_{c2}\left(T\right)/dT=3.86\;T/К$ was determined, which gives $H_{c2}^{WHH}\left(0\right)=51.3\;T$. The paramagnetic limit is *H_p_*(0) = 58.3 T, and exceeds the upper critical field $H_{c2}^{WHH}\left(0\right)$. From the slope of the upper critical field, one can estimate the coherence length $\xi_{0}\left(0\right)$ in the «pure limit» by using the following Equation (1):

(1) $$-dH_{c2}\left(T\right)/dT=\Phi_{0}/2\pi T_{c}\xi_{0}^{2}\left(0\right)$$

where $\Phi_{0}$ is the quant of magnetic flow. Using Equation (1), we found that the coherent length is $\xi_{0}\left(0\right)=24.9\;Å$ for the sample Ba(Fe_0.95_Ni_0.05_)_2_As_2_. According to the Ginzburg–Landau (GL) theory, the same length in the «dirty limit» is $\xi_{\mathrm{GL}}\left(0\right)=18.4\;Å$. For calculating some parameters, we used the Fermi velocity $v_{f}$, which for the Ni-doped Ba122 system takes into account the effective mass: $m^{*}=4m_{0}$ [25] is $v_{f}=2.33\times 10^{7}$ cm/s. We also estimated the coefficient of electronic specific heat, $\gamma_{n}$, which in the «dirty limit» has the following expression:

(2) γn=9×1011π3kb(−dHc2(T)/dT)Tc12ecρab

where $k_{b}$ is the Boltzmann constant, $\rho_{ab}$ is the film resistivity, e is the charge of the electron, and c is the speed of the light in vacuum. Estimating Equation (2) gives the following values: for Ba(Fe_1.95_Ni_0.1_)As_2_, $\gamma_{n}=27.2$ mJ/mol·K^2^, and for Ba(Fe_1.92_Ni_0.16_)As_2_, $\gamma_{n}=18.3$ mJ/mol·K^2^. The obtained values of $\gamma_{n}$ agree quite well with the experimental data for single crystals [26,27].

In addition, one can estimate the density of states at the Fermi level from the known value of $\gamma_{n}$, which for the Ba(Fe_0.95_Ni_0.05_)_2_As_2_ sample is *N*(0) = 2.83 × 10^22^ eV^−1^ cm^−3^, or N*(0) = 5.8 states/eV spin unit cell, and for Ba(Fe_0.92_Ni_0.08_)_2_As_2_ is *N*(0) = 1.89 × 10^22^ eV^−1^ cm^−3^ or N*(0) = 18.3 states/eV × spin unit cell. The values obtained for the density of states N*(0) agree quite well with the available data for single crystals [28,29]. In general, the values of the density of states at the Fermi level for the Ni-doped Ba122 system are quite low even in comparison with a HTSC’s materials with similar *T_c_* and resistivity (for example, the Nd_1.85_Ce_0.15_CuO_4_ system has *N*(0) ~ 5.76 × 10^22^ eV^−1^ cm^−3^ [30]) and in comparison with the ordinary superconductor (Nb_3_Sn *N*(0) ~ 25.63 × 10^22^ eV^−1^ cm^−3^ [17]). In addition, the lower critical field *H_c_*_1_ and the GL parameter $k_{\mathrm{GL}}$ were calculated. The results for the calculation of the electronic properties for both films are summarized and compared to the single crystal values in Table 2.

In general, there is good agreement if we compare the results of the magnetic properties for our thin films with single crystals. For example, Wang et al. [32] published a rather detailed study on the magnetic properties of Ba(Fe_1−x_Ni_x_)_2_As_2_ single crystals with different doping levels, and our results differ only slightly except for the mismatch of the paramagnetic limit *H_p_*(0) between the thin films and the single crystals. This is due to different approaches for the calculation of this parameter. In our calculations, we considered a gap value of Δ = 2.6 *k_B_T_c_*, which is consistent with experimental data for pnictides [24]. However, Rodière et al. [31] estimated the paramagnetic limit *H_p_*(0) based on the Bardeen–Cooper–Schrieffer (BCS) theory (Δ = 1.86 *k_B_T_c_*) and if we use the same approach, we get *H_p_*(0) = 39.7 T (Ba(Fe_0.95_Ni_0.05_)_2_As_2_) and *H_p_*(0) = 20.08 T (Ba(Fe_0.92_Ni_0.08_)_2_As_2_), which is in good agreement with the values for single crystals. Furthermore, some inconsistency between the results is mainly associated with the lower *T_c_* in single crystals compared to the obtained films, and with the structural inhomogeneities of the latter. Moreover, our calculations of *H_c_*_2_ and *H_p_* show that the orbital mechanism is mainly responsible for the pair breaking by a magnetic field in the obtained films, whereas the contribution of the spin-orbit interaction is negligible. Although *T_c_* and the resistivity *ρ* of the films are significantly different for the Ba(Fe_0.92_Ni_0.08_)_2_As_2_ and Ba(Fe_0.95_Ni_0.05_)_2_As_2_, no significant changes in the density of the states are observed. This behavior may be due to the presence of an electron phase separation in high-*T_c_* superconductors [34], i.e., the coexistence of both superconducting and non-superconducting regions, which occurs in a structurally perfect crystal with a small coherence length.

## 4. Conclusions

In summary, we performed a detailed study on the structural, superconducting, and electronic properties of Ba(Fe_1−x_Ni_x_)_2_As_2_ epitaxial thin films grown by pulsed laser deposition. Diffraction studies showed that the films have a single-crystal-like structure with a good crystalline quality, being only slightly inferior to single crystals. We confirmed that the unit cell of Ba(Fe_1−x_Ni_x_)_2_As_2_ epitaxial layers undergoes an additional tetragonal compression compared to single crystals. The magnetic susceptibility proved a sharp diamagnetic signal, whereas the *T_c_* is even slightly higher than the one for single crystals. The results of an approximation for the temperature dependence of the critical magnetic fields *H_c_*_2_ along the *c*-axis, using the WHH model, indicate that the pair breaking by a magnetic field is mainly due to the orbital mechanism in our films, whereas the contribution of the spin-orbit interaction is negligible. We evaluated the density of electronic states at the Fermi level, the electron-specific heat coefficient, and other electronic parameters of this compound, and their dependence on the dopant concentration in the framework of the GLAG theory. Despite the fact that the *T_c_* and resistivity *ρ* of the films are significantly different for Ba(Fe_0.92_Ni_0.08_)_2_As_2_ and Ba(Fe_0.95_Ni_0.05_)_2_As_2_, no significant changes in the density of states are observed.

## Figures and Tables

**Table 1:** Lattice parameters of the Ba(Fe_1−x_Ni_x_)_2_As_2_ thin films.

Lattice Parameters	Ni Concentration	
	x = 0.05	x = 0.08
*a* = *b*, Å	3.952	3.954
*c*, Å	13.0405	13.016
V, ${Å}^{3}$	204.599	204.343

**Table 2:** Electronic parameters of the Ba(Fe_1−x_Ni_x_)_2_As_2_ thin films and single crystals.

Parameter	x = 0.05 (Optimally Doped)		x = 0.08 (Overdoped)	
	Thin Film	Single Crystals	Thin Film	Single Crystals
Tc, K	21.1	20.3 [21,31]	10.3	10.9 [32]
$\rho_{ab}\times 10^{-5},\;\Omega \cdot \mathrm{cm}$	21.1	19–38.5 [27]	18	16 [27]
$-dH_{c2}\left(T\right)/dT,$ T/K	3.76	3.17 [32]	2.6	2.04 [32]
$H_{c2}^{WHH}\left(0\right),$ T	51.3	44.4–52 [31,32]	15.2	15.3–28 [31]
$H_{p}\left(0\right),$ T	58.3	38.1 * [32]	29.6	20.1 * [32]
$H_{c1}\;$× 10^−4^, T	192	180–210 [31]	109	80 [31]
$\lambda_{GL}$, nm	202	180–246 [10,31]	263	310 [10,31]
$\xi_{GL}$, Å	18.4	-	29.1	-
l, Å	35.1	-	57.3	-
N(0) × 10^22^, eV^−1^·cm^−3^	2.83	-	1.89	-
N*(0), states/eV·spin unit cell	5.85	~5.7 [29]	3.89	~4 [29]
$\gamma_{n}$, mJ/mol·K^2^	27.2	~24.6 [26,33]	18.3	~19 [26]
$k_{\mathrm{GL}}$	81.15	80 [31]	90.3	90 [31]

* In the text we consider the mismatch of $H_{p}\left(0\right)$ for thin films and single crystals.
